# Cyclophilin A in Arrhythmogenic Cardiomyopathy Cardiac Remodeling

**DOI:** 10.3390/ijms20102403

**Published:** 2019-05-15

**Authors:** Erica Rurali, Chiara Assunta Pilato, Gianluca Lorenzo Perrucci, Alessandro Scopece, Ilaria Stadiotti, Donato Moschetta, Michela Casella, Elisa Cogliati, Elena Sommariva, Giulio Pompilio, Patrizia Nigro

**Affiliations:** 1 Unit of Vascular Biology and Regenerative Medicine, Centro Cardiologico Monzino IRCCS, 20138 Milano, Italy; chiara.pilato@ccfm.it (C.A.P.); gianluca.perrucci@ccfm.it (G.L.P.); alessandro.scopece@ccfm.it (A.S.); ilaria.stadiotti@ccfm.it (I.S.); donato.moschetta@ccfm.it (D.M.); elena.sommariva@ccfm.it (E.S.); giulio.pompilio@ccfm.it (G.P.); patrizianigro@gmail.com (P.N.); 2 Cardiac Arrhythmia Research Centre, Centro Cardiologico Monzino IRCCS, 20138 Milano, Italy; michela.casella@ccfm.it; 3 Treviso Tissue Bank Foundation, 31100 Treviso, Italy; ecogliati@fbtv-treviso.org; 4 Dipartimento di Scienze Cliniche e di Comunità, Università degli Studi di Milano, 20126 Milano, Italy; 5 Department of Cardiovascular Surgery, Centro Cardiologico Monzino IRCCS, 20138 Milano, Italy

**Keywords:** cyclophilin A, arrhythmogenic cardiomyopathy, cardiac mesenchymal stromal cells, adipogenesis, fibrosis

## Abstract

Arrhythmogenic cardiomyopathy (ACM) is a genetic disorder characterized by the progressive substitution of functional myocardium with noncontractile fibro-fatty tissue contributing to ventricular arrhythmias and sudden cardiac death. Cyclophilin A (CyPA) is a ubiquitous protein involved in several pathological mechanisms, which also characterize ACM (i.e., fibrosis, inflammation, and adipogenesis). Nevertheless, the involvement of CyPA in ACM cardiac remodeling has not been investigated yet. Thus, we first evaluated CyPA expression levels in the right ventricle (RV) tissue specimens obtained from ACM patients and healthy controls (HC) by immunohistochemistry. Then, we took advantage of ACM- and HC-derived cardiac mesenchymal stromal cells (C-MSC) to assess CyPA modulation during adipogenic differentiation. Interestingly, CyPA was more expressed in the RV sections obtained from ACM vs. HC subjects and positively correlated with the adipose replacement extent. Moreover, CyPA was upregulated at early stages of C-MSC adipogenic differentiation and was secreted at higher level over time in ACM- derived C-MSC. Our study provides novel ex vivo and in vitro information on CyPA expression in ACM remodeling paving the way for future C-MSC-based mechanistic and therapeutic investigations.

## 1. Introduction

Arrhythmogenic cardiomyopathy (ACM) is a rare inherited disease characterized by progressive cardiomyocyte death and fibro-fatty replacement of functional myocardial tissue [1,2]. The typical form of ACM is due to mutations in genes encoding for desmosomal proteins [3,4]. Alterations in desmosome organization compromise the mechanical and electrical interaction among cardiac cells [5,6], especially under conditions that increase myocardial strain (e.g., strong athletic activity), and provide signaling at the basis of the widely recognized ACM pathological features (i.e., fibrosis, adipogenesis, and inflammation) [7,8,9,10,11]. The progression of fibro-fatty substitution, predominantly located in the right ventricle (RV), causes ventricular dilatation, wall thinning, and in about 50% of patients, aneurysmal dilation typically located in the “triangle of dysplasia” (i.e., RV outflow tract, apex, and infundibulum) [12]. Moreover, this remodeling process negatively influences the already compromised intraventricular conduction of electrical impulses, worsening ventricular arrhythmias and increasing the risk of sudden cardiac death [5,13,14]. Despite several theories on the possible cellular and molecular pathways involved in ACM pathogenesis have been postulated in the last few years [13,15,16], the exact mechanisms are not fully unraveled. Noteworthy, we have recently demonstrated that cardiac mesenchymal stromal cells (C-MSC) contribute to adipogenesis in ACM patients [17] introducing a new tool for ACM in vitro modeling [18]. 

Cyclophilin A (CyPA) is a ubiquitous immunophilin that physiologically regulates protein folding, trafficking, and interaction [19]. However, it is also involved in pathological processes underlying several cardiovascular diseases (e.g., cardiac hypertrophy, inflammatory cardiomyopathies, critical limb ischemia, and coronary artery diseases) [20,21,22,23,24,25,26]. Of note, CyPA can be secreted by endothelial cells, vascular smooth muscle cells, cardiac fibroblasts, and inflammatory cells to exert its autocrine and paracrine actions [27]. In cardiac hypertrophy, CyPA promotes cardiac fibroblast proliferation and migration, while in atherosclerosis it participates both to fatty streak formation and to low-density lipid uptake in the vessel wall [21,28]. Interestingly, CyPA has been recently proposed as a novel adipokine due to its proadipogenic activity demonstrated both in vitro in 3T3-L1 and in vivo in an obese murine model [29]. 

Thus, considering the role of CyPA in the aforementioned processes, we have investigated CyPA modulation in ACM. Interestingly, we found that CyPA expression (1) was upregulated in ACM patient-derived tissue samples, (2) correlated with tissue fatty substitution extent, and (3) was modulated in patient-derived C-MSC subjected to adipogenic stimuli.

## 2. Results

### 2.1. CyPA Expression in the RV Tissue

**Figure 1:**
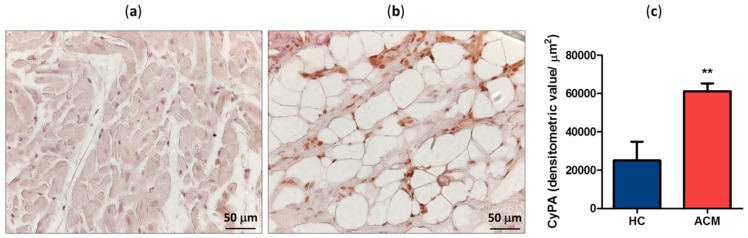
Representative cyclophilin A (CyPA) immunostaining of healthy control (HC) (**a**) and arrhythmogenic cardiomyopathy (ACM) patient (**b**) right ventricle (RV) sections. Positive signal stained brown. Quantification of CyPA expression level in HC and ACM RV tissues (**c**). ** *p* value ≤ 0.01 at Student’s *T* test.

CyPA expression levels have been evaluated by immunohistochemistry in RV specimens derived from three ACM patients and four healthy controls (HC). CyPA expression levels, calculated as densitometric value normalized for the analyzed tissue area, were significantly higher in the tissue samples of ACM (mean ± SEM: 61,135 ± 2348) than in those of HC (24,998 ± 4911, *p* = 0.002; Figure 1a–c). Interestingly, differences in CyPA distribution have been observed in the RV sections of ACM patients with respect to HC. Indeed, in HC RV tissue CyPA was localized predominantly at nuclear level, while in ACM samples evenly accumulation was observed at nuclear as well as cytosolic and extracellular matrix level (Figure 1a,b).

### 2.2. Correlation between CyPA Expression Levels and Fatty Substitution

**Figure 2:**
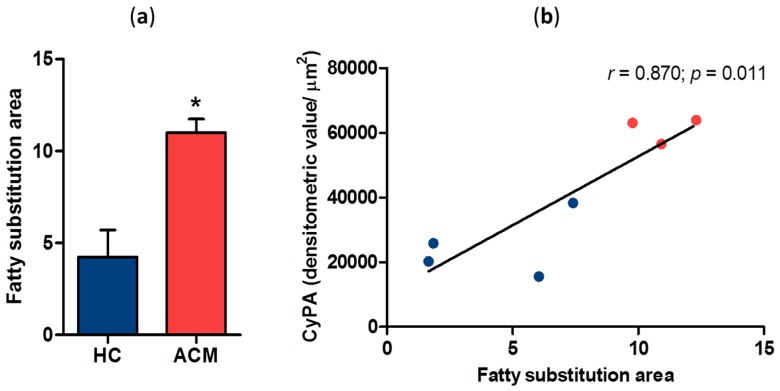
Quantification of fatty substitution area in HC and ACM RV sections (**a**). Correlation between CyPA expression levels and the extent of fatty substitution (**b**). * *p* value ≤ 0.05 at Student’s *T* test.

To investigate whether CyPA expression levels correlate with myocardial fatty substitution, we assessed in RV the amount of myocardial area replaced by adipose tissue. Noteworthy, fatty substitution was significantly larger in ACM patients than in HC. In particular, fatty substitution area in the RV sections corresponded to 4.2 ± 2.9 of analyzed tissue in HC and 11.0 ± 1.3 in ACM patients (*p* = 0.014, Figure 2a). Interestingly, CyPA expression levels significantly and positively correlated with adipose substitution extent in RV sections (Pearson *r* = 0.870, *p* = 0.011; Figure 2b).

Then, to elucidate whether the increased CyPA expression found in ACM patients was principally related to the disease or to the presence of adipocytes, we assessed CyPA expression levels in a subset of selected RV tissue fields obtained from an aged HC, who showed physiological cardiac adipose tissue accumulation, and from an ACM patient. Specifically, we selected the fields characterized mostly by intact myocardium or mostly by the presence of adipocytes (Figure S1a–d). Interestingly, we found that CyPA was overall more expressed in ACM- than in HC-derived RV tissue fields (Figure S1e). Moreover, it was higher in peri-adipocyte than in myocyte regions both in HC and in ACM patients (Figure S1e). Together, these results suggest a specific association between CyPA modulation and the ACM disease process underlying fatty substitution.

We then performed a Masson’s trichrome staining to evaluate the amount of fibrotic tissue in collected slides. As expected, fibrotic substitution was significantly larger in ACM patients than in HC (Figure S2a,b). In particular, fibrotic deposition area was 0.05 ± 0.02 in HC and 1.70 ± 0.86 in ACM patients (*p* = 0.010, Figure S2c). Anyway, CyPA expression levels only partially correlated with connective tissue deposition (Spearman *r* = 0.750, *p* = 0.066; Figure S2d).

### 2.3. CyPA Is Expressed in C-MSC during Adipogenic Differentiation

**Figure 3:**
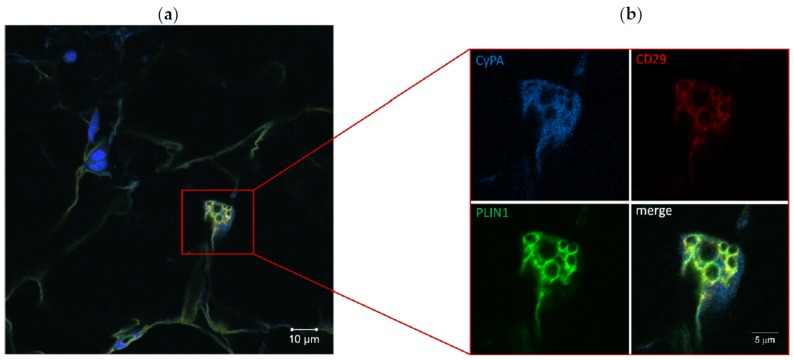
Immunofluorescence staining of ACM RV section (**a**). Particular of a preadipocyte, positive for CD29 and perilipin 1 (PLIN1), expressing cyclophilin A (CyPA) (**b**). The image of merged signals is also shown.

To evaluate whether CyPA was expressed by C-MSC during adipogenesis, we performed an immunofluorescence staining on ACM RV sections with three specific antibodies recognizing CyPA, the mesenchymal marker CD29, and the adipocyte marker perilipin 1 (PLIN1, a protein localized at the membrane of lipid droplets). The triple staining clearly revealed that those cells found double positive for CD29 and PLIN1, representing C-MSC at a preadipocyte differentiation stage, also expressed CyPA in the cytoplasm (Figure 3). 

### 2.4. CyPA Expression is Modulated during C-MSC Adipogenic Differentiation

**Figure 4:**
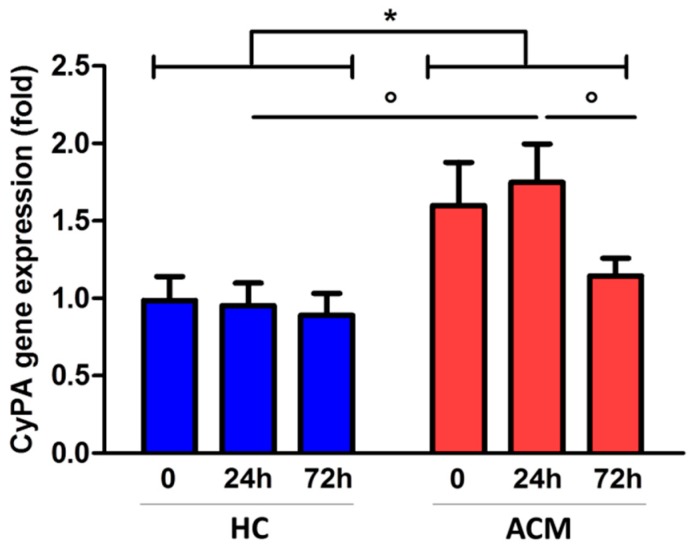
*CyPA* gene expression levels in HC- and ACM-derived cardiac mesenchymal stromal cells (C-MSC) cultured in adipogenic condition. * *p* value ≤ 0.05 at two-way ANOVA and ° *p* value ≤ 0.05 at Bonferroni post-test.

We then isolated C-MSC from ACM patients and HC RV specimens and cultured them in adipogenic condition to evaluate *CyPA* gene expression levels at different time points (0, 24 h, and 72 h). Interestingly, C-MSC derived from ACM and HC showed a different pattern of *CyPA* gene expression over time (*p* = 0.021, Figure 4). Specifically, *CyPA* gene expression was modulated over time in ACM C-MSC, but not in HC C-MSC. The highest difference was observed after 24 h of adipogenic conditioning, when *CyPA* gene expression was upregulated in ACM (mean ± SEM: 1.75 ± 0.25) with respect to HC C-MSC (0.95 ± 0.15, *p* = 0.015; Figure 4). 

**Figure 5:**
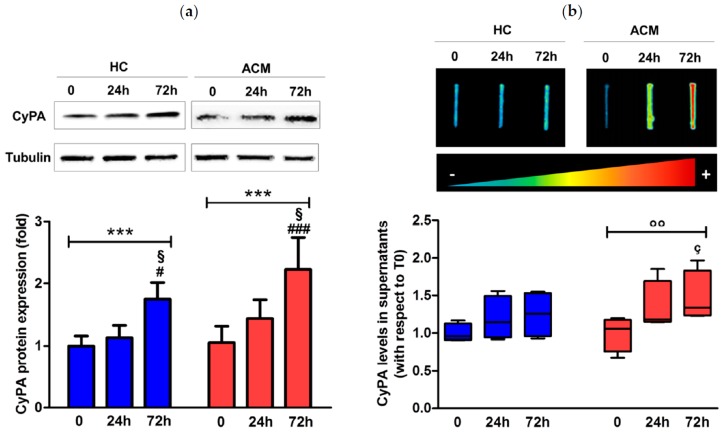
CyPA protein expression levels in cell lysates (**a**) and supernatants (**b**) of HC and ACM C-MSC cultured in adipogenic condition for 72 h. *** *p* value ≤ 0.001 at two-way ANOVA and *§ p* value ≤ 0.05 vs. “24 h”, # *p* value ≤ 0.05 vs. “0”, ### *p* value ≤ 0.001 vs. “0” at Bonferroni post-test. °° *p* value ≤ 0.01 at Friedman test, and *ç p* value ≤ 0.05 vs. “0” at Dunn’s Multiple Comparison post-test.

Furthermore, we evaluated CyPA amount in cell lysates by Western blot analysis. CyPA protein expression levels increased over time in ACM C-MSC (mean ± SEM: 1.05 ± 0.26 at baseline, 1.44 ± 0.30 at 24 h, and 2.23 ± 0.51 at 72 h, *p* ≤ 0.001) as well as in HC C-MSC (1 ± 0.15 at baseline, 1.13 ± 0.20 at 24 h, and 1.75 ± 0.27 at 72 h, *p* ≤ 0.001) with a significant peak of increase at 72 h (Figure 5a). 

Since CyPA can be secreted from different cell types, we evaluated, by slot blot analysis, the protein amount in the supernatants of C-MSC culture during adipogenic differentiation finding a significant increase in CyPA amount only in the media of ACM C-MSC over time (median interquartile range (IQR): 1.06 (0.76–1.18) at baseline, 1.18 (1.15–1.69) at 24 h, and 1.34 (1.24–1.83) at 72 h, *p* ≤ 0.01) and specifically after 72 h of treatment vs. baseline (*p* ≤ 0.05, Figure 5b).

## 3. Discussion

The data obtained in this study support the hypothesis of a possible involvement of CyPA in ACM cardiac remodeling. First of all, we found higher CyPA expression levels in ACM than in HC RV tissue. Then, we pointed out a positive correlation between the RV adipose tissue extent and the expression levels of CyPA. As expected, we also detected a moderate degree of adipose tissue in HC specimens of the RV, probably due to a physiological age- and body weight-dependent accumulation [30]. However, in ACM patient-derived RV tissue we found an extensive myocardial fatty substitution in parallel with a considerable expression of CyPA, principally located in the adipose regions, suggesting an association between CyPA levels and the ACM tissue degenerative process. The link between CyPA and adipogenesis is supported by previously reported evidences in two different pathological contexts, such as atherosclerosis and obesity [28,29]. Specifically, CyPA depletion in ApoE^−/−^ mice determined a decreased atherosclerotic lesion burden, owing to an impaired regulation of scavenger receptors that determines fewer low-density lipid uptake into the vessel wall [28]. Furthermore, the lack of CyPA determined impairment in lipid accumulation both in an obese mouse model and in an already adipo-committed cell line (3T3-L1) [29]. These evidences indicate that the role of CyPA in adipogenesis is not specifically confined to the cardiac district.

Thus, to further investigate CyPA involvement in cardiac ACM adipogenic differentiation, we took advantage of C-MSC, a novel in vitro tool that we have recently demonstrated to recapitulate ACM features [17]. Interestingly, we observed that CyPA was expressed in ACM-derived C-MSC during adipogenic differentiation, both ex vivo by immunofluorescence and in vitro in the presence of adipogenic conditioning. Specifically, CyPA transcript was modulated over the three days of adipogenic stimuli only in ACM-derived C-MSC, while protein production increased over time both in ACM- and HC-derived C-MSC cultured in adipogenic medium. Furthermore, we highlighted that only patient-derived C-MSC released increasing amount of CyPA over time. This finding is in agreement with previous evidences indicating CyPA as a novel adipokine secreted during 3T3-L1 adipocyte differentiation [31]. Altogether, our results suggest that ACM-derived C-MSC, genetically more prone to accumulate lipids [17], generally respond to the adipogenic stimuli with an increased CyPA production and secretion. Since CyPA has been found to be strongly involved in the active recruitment of leukocytes, macrophages, and T cells in different pathological contexts [19,25,32,33,34], we speculate that, through a paracrine/autocrine action, it may promote the ACM proinflammatory milieu [11] which has been demonstrated to boost the typical cardiac remodeling [10].

In conclusion, although our current findings are observational and thus do not demonstrate a mechanistic link between CyPA modulation and genetic-related ACM defects, they turn the spotlight on CyPA as a player in the detrimental pathological context of ACM. Thus, we envisage CyPA as a future object of C-MSC-based translational investigations to fill this gap of knowledge.

## 4. Materials and Methods

### 4.1. Sample Collection

This study complies with the Declaration of Helsinki and was approved by “Centro Cardiologico Monzino IRCCS” Ethics Committee (07/06/2012). Written informed consent was obtained from all participants. Bioptic samples of RV tissue were collected during endomyocardial mapping procedures [35] from 9 patients with clinical suspicion of ACM. The clinical diagnosis of ACM was confirmed following the standard criteria reported in the 2010 modified Task Force Criteria [36]. Out of the 9 collected bioptic specimens, 3 were embedded in paraffin and processed for tissue slide sectioning, while the other 6 were digested in order to isolate C-MSC, as previously reported [37]. As control, tissues and C-MSC were obtained from the RV endomyocardial samples of 7 cadaveric donors, who died accidentally. The latter were provided by the Treviso Tissue Bank Foundation (Treviso, Italy). 

### 4.2. Immunohistochemistry

To perform CyPA immunostaining, slice sections were deparaffinized, rehydrated, and boiled for 20 min in the target retrieval solution (sodium citrate, pH 6.0; DAKO, Glostrupt, Denmark). After washing in phosphate-buffered saline (PBS) supplemented with 0.1% Tween-20 (PBST), slides were incubated in 3% hydrogen peroxide for 10 min and blocked in PBST with 5% goat serum (1 h, room temperature, RT). Primary antibody against human CyPA (Bioss, Woburn, MA, USA) was dissolved in antibody diluent (DAKO) and incubated in a humidified chamber (O/N, 4 °C). Sections were incubated first with biotin-conjugated goat anti-rabbit antibody (Vector Laboratories, Burlingame, CA, USA) and then with HRP-conjugated streptavidin (ABC kit) (Vector Laboratories) for 30 min, RT. Immunoreactions were revealed using 3,3-diaminobenzidine (ImmPACT DAB substrate) (Vector Laboratories) as chromogen, and slides were counterstained with hematoxylin. Negative controls were performed omitting primary antibody incubation. Quantification of CyPA positive staining and of fatty substitution area in the RV sections was made by taking images with an Axioskop II microscope (Zeiss, Oberkochen, Germany) and using AxioVision 4.8.1 software (Zeiss). Twenty different fields from each section were taken at 20× magnification for each staining. CyPA expression levels were defined as the fraction of the positive staining quantified in the entire RV bioptic sample, expressed in densitometric value, to the total evaluated area (µm^2^), while fatty substitution extent was expressed as the adipose area adjusted for the field area.

### 4.3. Immunofluorescence

For immunofluorescence staining, RV tissue sections were deparaffinized, rehydrated, and boiled for 20 min in target retrieval solution (Tris-EDTA, pH 9.0; DAKO). After washing in PBS, slides were blocked for 30 min in 10% goat serum-PBS (Sigma Aldrich, St. Louis, MO, USA) and incubated with specific primary antibodies against CyPA (1:100, sc-133494; Santa Cruz Biotechnology, Santa Cruz, CA, USA), PLIN1 (1:100, BP5015; OriGene, Herford, Germany) and CD29 (1:200, NCL-CD29; Leica, Wetzlar, Germany) in 2% goat serum-PBS O/N at 4 °C. After washing in PBS, sections were incubated with Alexa546 (ThermoFisher, Waltham, MA, USA), Alexa488 (Santa Cruz Biotechnology), and Alexa633 (ThermoFisher) fluorochrome conjugated secondary antibodies dissolved in 2% goat serum-PBS (1 h, RT in the dark), respectively. To visualize cell nuclei, Hoechst33342 (Invitrogen, Carlsbad, CA, USA) diluted 1:1000 in PBS (15 min, RT in dark) was added. Twenty images for each section were taken at 20× magnification using the software Zen2010D of the confocal microscope (Zeiss LSM710-ConfoCor 3 LSM). 

### 4.4. C-MSC Culture

The C-MSC were cultured in growing medium (TMES: Iscove’s Modified Dulbecco’s Medium [IMDM], 20% fetal bovine serum [FBS], 0.02 M Glutamine, 10,000 U/mL Penicillin, 10,000 µg/mL Streptomycin, 10 ng/mL basic fibroblast growth factor) or adipogenic conditioning (ADIPO: IMDM, 10% FBS, 0.02 M Glutamine, 10,000 U/mL Penicillin, 10,000 µg/mL Streptomycin, 0.5 mM 3-isobutyl-1-methylxanthine, 1 µM hydrocortisone, 0.1 mM indomethacin) for 3 days on the basis of experimental design. 

### 4.5. qRT-PCR

Total RNA was extracted from HC and ACM C-MSC using the Total RNA Purification Plus kit (Norgen Biotek Corp., Thorold, ON, Canada) and reverse transcribed by SuperScript III First-Strand Synthesis SuperMix for qRT-PCR (Invitrogen). Then qRT-PCR was performed in triplicate using 15 ng of cDNA and the iTaq Universal SYBR Green Supermix (Bio-Rad Laboratories, Hercules, CA, USA). All these processes were performed following manufacturers’ instructions. The primer sequences of analyzed genes are reported in Table 1.

### 4.6. Western Blot

Cytoplasmic extracts of C-MSC were collected by cell lysis buffer (Cell Signaling Technology, Danvers, MA, USA) supplemented with a protease inhibitor cocktail (Sigma Aldrich) and quantified by Bio-Rad Protein Assay (Bio-Rad). Then, equal amount of total protein lysates were subjected to reducing SDS-PAGE (Novex 4–12% Tris-glycine Mini Gels, Bio-Rad Laboratories) and transferred at 25 V, 1.3 A for 10 min onto a nitrocellulose membrane by Trans-Blot turbo blotting system (Bio-Rad Laboratories). After blocking with 5% non-fat dry Blotto milk (ChemCruz Huissen, The Netherlands) in washing buffer (0.1% Tween-20 in TBS), the membrane was incubated O/N at 4 °C with the appropriate primary antibody. The primary antibodies used were specific for CyPA (Santa Cruz Biotechnology) and tubulin (Sigma Aldrich). The membrane was then incubated with the appropriate peroxidase-conjugated secondary antibodies for 1 h and developed using enhanced chemiluminescence detection systems (Thermo Scientific, Rockford, IL, USA). The images were acquired and quantified respectively with the Alliance Mini 2M and the Alliance Mini 4 16.07 software (UVITEC, Cambridge, UK). The amount of CyPA was normalized to housekeeping protein tubulin.

### 4.7. Statistical Analysis

Data are presented as mean ± standard error of the mean (SEM) or median (25–75 percentile), as appropriate. The Shapiro-Wilk test was used to assess the normal distribution of the considered variables. Comparison between two groups was made using the Student’s *T* or the Mann-Whitney U test, as appropriate. For analyses over time, two-way ANOVA for repeated measures with Bonferroni post-test or Friedman test with Dunn’s Multiple Comparison post-test have been performed, as appropriate. Correlation analyses were performed by the Pearson or the Spearman’s rank correlation test, as appropriate. Statistical significance was set at *p* ≤ 0.05. All the analyses were performed using GraphPad Prism 5 (GraphPad Software, San Diego, CA, USA).

## Figures and Tables

**Table 1:** Primer sequences (5′-3′).

Gene	Forward Primer	Reverse Primer
*CyPA*	CCA CCG TGT TCT TCG ACA TT	CCT TGT CTG CAA ACA GCT CA
*GAPDH*	ATG TTC GTC ATG GGT GTG AA	GTC TTC TGG GTG GCA GTC AT

## Supplementary Materials

Supplementary materials can be found at https://www.mdpi.com/1422-0067/20/10/2403/s1.

## Abbreviations

ACM	Arrhythmogenic cardiomyopathy
C-MSC	Cardiac mesenchymal stromal cells
CyPA	Cyclophilin A
GAPDH	Glyceraldehyde 3-phosphate dehydrogenase
HC	Healthy control
IQR	Interquartile range
PLIN1	Perilipin 1
RV	Right Ventricle
