# Supplementary material for: Cyclophilin A in Arrhythmogenic Cardiomyopathy Cardiac Remodeling
Source: Int J Mol Sci. 2019 May 15;20(10):2403. doi: 10.3390/ijms20102403 (PMC6566687; doi:10.3390/ijms20102403)
Supplement: Supplementary file 1 [file ijms-20-02403-s001.pdf]

International Journal of *Molecular Sciences*

MDPI

Supplementary Figure 1

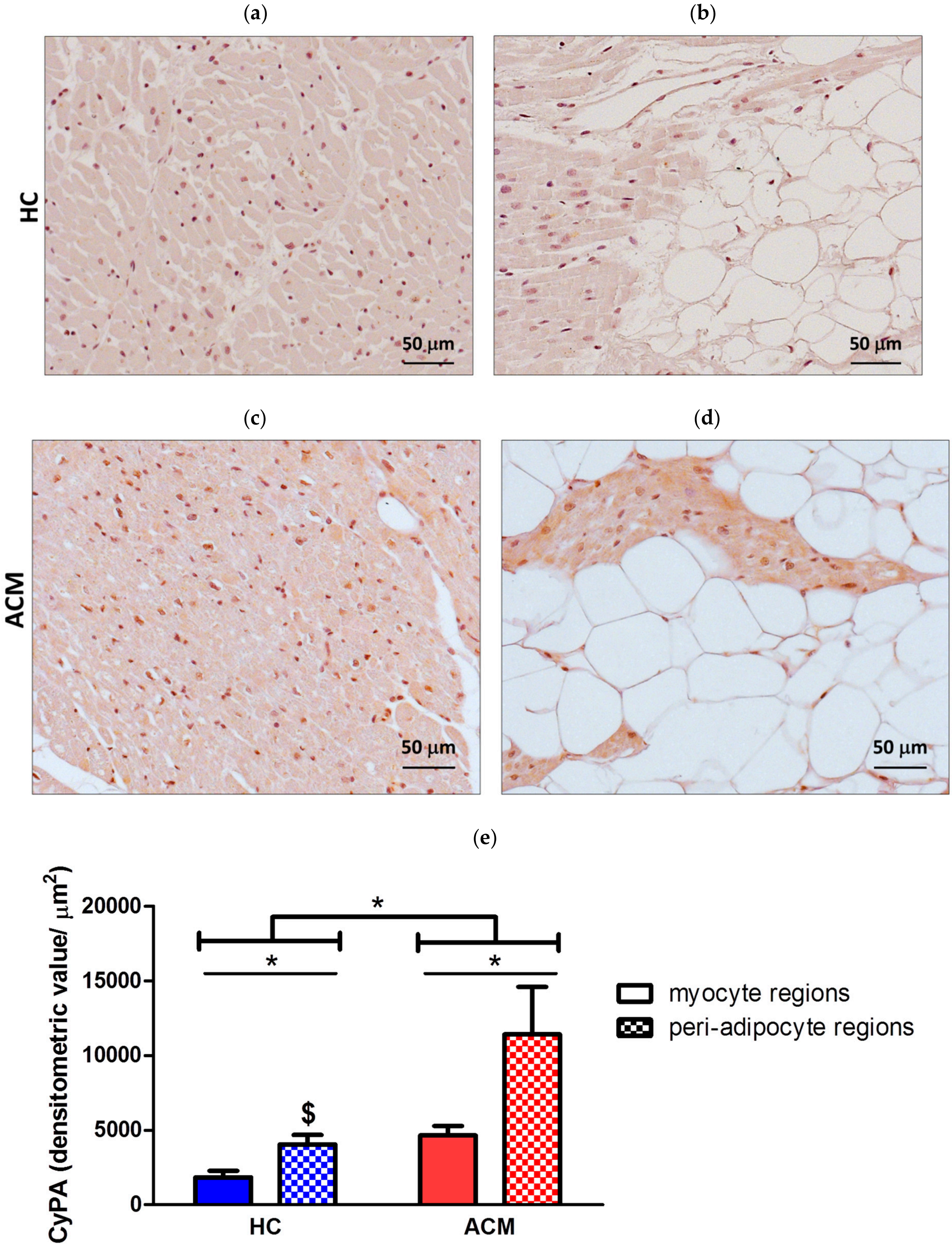

Figure S1. CyPA immunostaining of selected regions characterized by the presence of mainly myocytes or adipocytes in the RV sections obtained from an aged HC((**a**) and (**b**), respectively) and an ACM patient ((**c**) and (**d**), respectively). Positive signal stained brown. Quantification of CyPA expression levels in the selected regions (**e**). *p value ≤0.05 at two-way ANOVA, $ p value ≤0.05 vs. ACM peri-adipocyte region at Bonferroni post-test.

Supplementary Figure 2

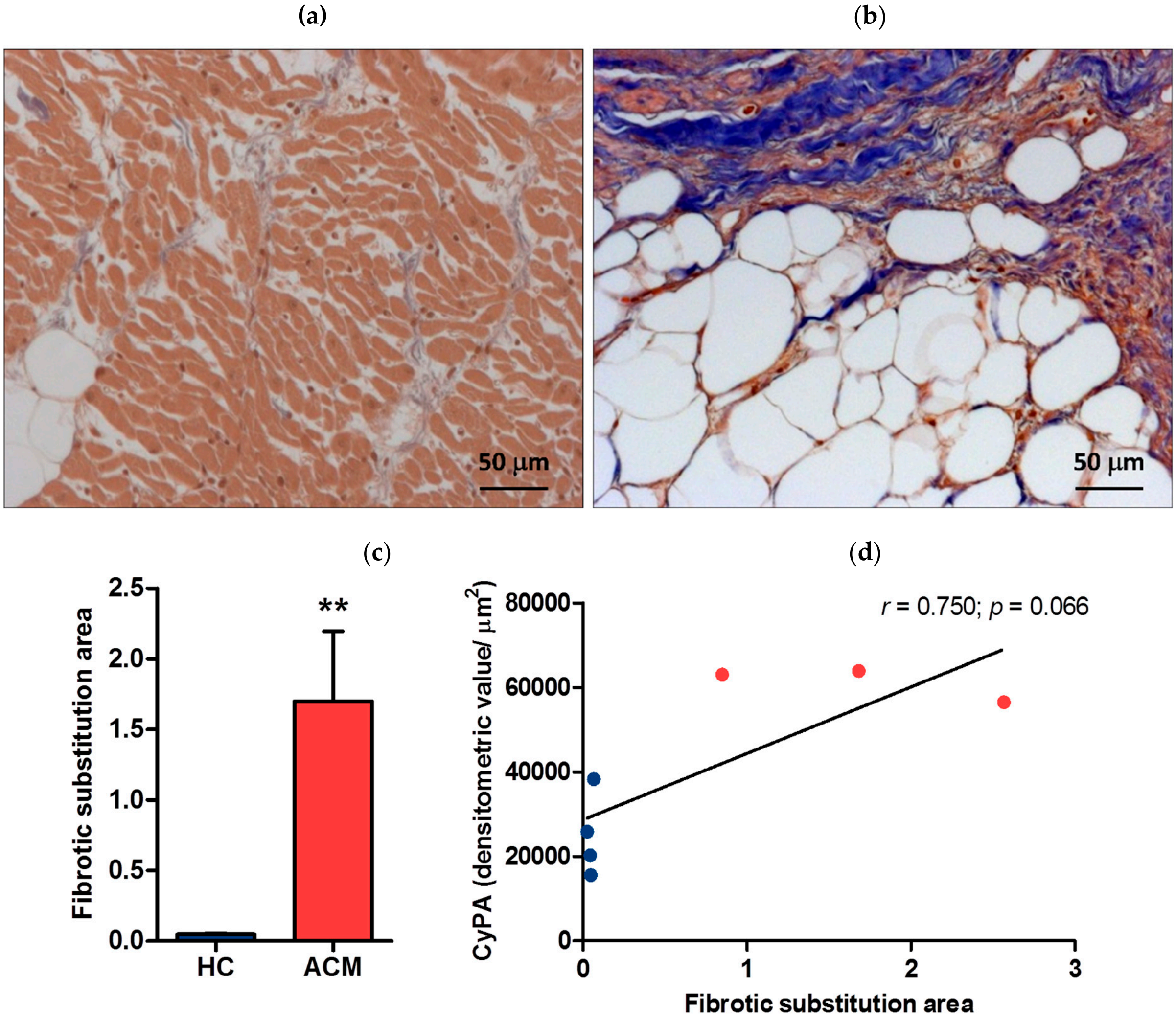

Figure S2. Representative Masson's trichrome staining of HC (**a**) and ACM (**b**) RV tissue. Connective tissue is stained in blue. Quantification of fibrotic substitution area in HC and ACM RV sections (**c**). Correlation between CyPA expression levels and the extent of fibrotic substitution (**d**). ** p value ≤0.01 at Student's T test.

Supplementary Materials and Methods

Masson's trichrome staining

To stain fibrotic tissue in histological samples derived from 3 ACM and 4 HC, Masson's trichrome staining kit (Bio-Optika) was exploited. Slice sections were deparaffinized maintaining them in xylene for 6 min and subsequently they were hydrated through descending alcohols (from 100% ethanol to 50% ethanol) for 3 min in each passage. The tissues were stained following the product datasheet. After removing the excessive colorant, the slices were dehydrated through ascending alcohols and cleared in xylene. Quantification of fibrotic substitution area (blue signal) in the RV sections was made by taking images with an Axioskop II microscope (Zeiss) and by using AxioVision 4.8.1 software (Zeiss). Twenty different fields from each section were taken at 20× magnification. Fibrotic substitution was expressed as the ratio of the positive Masson's area and the total analyzed tissue.
